# High Fat Diet Induces Liver Steatosis and Early Dysregulation of Iron Metabolism in Rats

**DOI:** 10.1371/journal.pone.0066570

**Published:** 2013-06-21

**Authors:** Rosaria Meli, Giuseppina Mattace Raso, Carlo Irace, Raffaele Simeoli, Antonio Di Pascale, Orlando Paciello, Teresa Bruna Pagano, Antonio Calignano, Alfredo Colonna, Rita Santamaria

**Affiliations:** 1 Department of Pharmacy, University of Naples Federico II, Naples, Italy; 2 Department of Veterinary Medicine and Animal Productions, University of Naples Federico II, Naples, Italy; Sapienza University of Rome, Italy

## Abstract

This paper is dedicated to the memory of our wonderful colleague Professor Alfredo Colonna, who passed away the same day of its acceptance. Fatty liver accumulation, inflammatory process and insulin resistance appear to be crucial in non-alcoholic fatty liver disease (NAFLD), nevertheless emerging findings pointed an important role also for iron overload. Here, we investigate the molecular mechanisms of hepatic iron metabolism in the onset of steatosis to understand whether its impairment could be an early event of liver inflammatory injury. Rats were fed with control diet or high fat diet (HFD) for 5 or 8 weeks, after which liver morphology, serum lipid profile, transaminases levels and hepatic iron content (HIC), were evaluated. In liver of HFD fed animals an increased time-dependent activity of iron regulatory protein 1 (IRP1) was evidenced, associated with the increase in transferrin receptor-1 (TfR1) expression and ferritin down-regulation. Moreover, ferroportin (FPN-1), the main protein involved in iron export, was down-regulated accordingly with hepcidin increase. These findings were indicative of an increased iron content into hepatocytes, which leads to an increase of harmful free-iron also related to the reduction of hepatic ferritin content. The progressive inflammatory damage was evidenced by the increase of hepatic TNF-α, IL-6 and leptin, in parallel to increased iron content and oxidative stress. The major finding that emerged of this study is the impairment of iron homeostasis in the ongoing and sustaining of liver steatosis, suggesting a strong link between iron metabolism unbalance, inflammatory damage and progression of disease.

## Introduction

The progression of non-alcoholic fatty liver disease (NAFLD) from a benign pathology as steatosis to more severe forms of liver diseases (i.e. hepatitis, fibrosis, cirrhosis, hepatocarcinoma) is determined by many factors or “hits” which may act in sequence or parallel, as recently proposed [1]. Indeed, fatty liver accumulation, inflammatory process and insulin resistance (IR) appear to be crucial in the onset of NAFLD, nevertheless emerging findings pointed an important role also for iron overload [2]. In fact, an association between IR and mild hepatic iron accumulation has been found particularly in patients with NAFLD [3], [4]. Iron deposits are found in hepatocytes, and/or Kupffer/sinusoidal cells, promoting cell damage [5]. Iron cytotoxicity is due to the ability by which free iron, as Fe^2+^ ions, participates in redox reactions, leading to the production of harmful oxygen radicals that can damage cellular structures [6]. To suppress the potential deleterious effects of iron, cells have evolved homeostatic mechanisms that regulate transport, storage and mobilization of this element. At cellular level, maintenance of iron homeostasis is largely accomplished by the transferrin receptor -1 (TfR-1), which allows iron uptake, and by ferritin, which is crucial to sequester this metal in a non-toxic form [7]. The levels of these and other proteins involved in iron metabolism are mainly regulated post-transcriptionally by interaction between the iron regulatory proteins (IRP1 and IRP2) and stem-loop structures, termed iron responsive elements (IREs), located in the 5′-untranslated region (UTR) of ferritin mRNAs and in the 3′-UTR of TfR mRNA [8], [9]. Intracellular free iron regulates IRP1, affecting its RNA-binding affinity, and IRP2, inducing its proteasomic degradation [10], [11]. In particular, in iron-depleted cells, IRPs strongly interact with IREs within the 3′-UTR of TfR-1 mRNA, increasing mRNA half-life and TfR-1 protein expression [12]. Simultaneously, the binding of IRPs to IRE in the 5′-UTR of ferritin mRNA prevents its synthesis [13]. On the contrary, IRPs affinity to IREs is low in iron-repleted cells, resulting in rapid TfR-1 mRNA degradation and in efficient translation of ferritin. Besides the intracellular iron concentration, IRPs RNA binding activity is also regulated by iron independent factors, such as oxidative stress [14], nitric oxide signalling [15], viral infection [16], hypoxia/reoxygenation [17], [18] and estrogens [19].

Systemic iron homeostasis is tightly controlled by hepcidin. This peptide hormone, predominantly expressed in the liver [20], [21], modulates iron availability promoting the internalization and degradation of the cellular iron exporter ferroportin-1 (FPN-1). In the liver the expression of hepcidin is increased dramatically in inflammation and because of chronic diseases associated to hypoferremia [22]. In particular, among proinflammatory cytokines, IL-6 is thought to be central to this mechanism [23]. Although increased liver iron deposits have been reported in patients with NAFLD [24], [25], it has not yet been established the mechanisms of iron overload, and whether alterations of iron metabolism contributes to the onset of liver disease and its progression. Actually, the influence of iron on the development of NAFLD has been the object of many experimental and clinical studies, where the dysregulation of iron overload could appear crucial in a progressive disease phenotype [26]–[28].

Overnutrition, the central feature of the “modern lifestyle”, with either carbohydrates (fructose and sucrose) or fats (fatty acids and cholesterol) or both has a key role in the multiple parallel hits-related to NAFLD. In this study rats fed a high fat diet, representing a model of hepatic steatosis and IR, were used to investigate whether the dysregulation of hepatic iron metabolism plays a role in the early events of steatosis and inflammation and explored the potential link between impaired iron homeostasis, liver inflammatory damage and ongoing of disease.

## Materials and Methods

### Ethics Statement

This study was carried out in strict accordance with the Institutional Guidelines and complied with the Italian D.L. no.116 of January 27, 1992 of Ministero della Salute and associated guidelines in the European Communities Council Directive of November 24, 1986 (86/609/ECC). All animal procedures reported herein were approved by the Institutional Animal Care and Use Committee (CSV) of University of Naples “Federico II” under protocol no. 2008-0099793. Prior to sample collection, animals were euthanized by an intraperitoneal injection of a cocktail of ketamine/xylazine, followed by cervical dislocation to minimize pain.

### Diets

Control nonpurified pelleted diet (Std, Global diet 77 2018) was purchased from Harlan Laboratories (Udine, Italy), and has 17% of energy derived from fat, 23% from protein, and 60% from carbohydrate. High-fat diet, purchased from Laboratorio Dottori Piccioni (Gessate, Milan, Italy), had 58,1% of energy derived from fat, 16% from protein, and 25.5% from carbohydrates. The composition of high fat diet has been previously described by Surwit et al. [29]. Control and high fat diets contained 3.30 and 5.56 kcal/g, respectively. Iron content was identical in the control and high-fat diet.

### Rat Model

After weaning, young male Sprague-Dawley rats (113.5±1.1 g; Harlan Laboratories), were randomly divided and fed with the control diet (CD; n = 8) or a high fat diet (HFD) for 5 or 8 weeks (n = 6 each group). This HFD rich in saturated and unsaturated fats and low in carbohydrates reproduces the pathogenetic and morphological events of IR and NAFLD [30]. Since no significant differences were evidenced between 5 and 8 weeks control groups in hepatic and biochemical parameters, a single control group was considered.

After 5 or 8 weeks treatment, all rats were anesthetized and, before sacrifice, blood was collected by cardiac puncture and serum obtained.

Throughout the treatment period, body weight was monitored once a week. At the end of both experimental periods bioelectrical impedance analysis was applied to body composition assessment at 5 and 8 weeks using a BIA 101 analyzer, modified for the rat (Akern, Florence, Italy),to determine fat mass, as previously described [31].

### Liver Preparation and Histological Analysis

Livers were excised, weighed and immediately frozen or fixed in neutered buffer formalin and mounted in OCT compound. Ten micrometer sections were stained with hematoxylin-eosin and Oil red O stains for the morphological and intra hepatocytes lipid evaluation. Steatosis was graded on a scale of 0–3 as follows: grade 0, absence of steatosis; grade 1<30% of hepatocytes affected; grade 2, 30–70% of hepatocytes affected; grade 3>70% of hepatocytes affected.

Intra-hepatocytes iron was assessed by Perls’ Prussian blue staining, according to Scheuer [32].

### Serum and Tissue Parameters

Aspartate amino transferase (AST), alanine amino transferase (ALT), triglycerides, high-density lipoprotein (HDL), low-density lipoprotein (LDL), triglycerides (TGL), iron, ferritin total iron binding capacity (TIBC), and transferrin saturation (%) were measured or calculated by standard procedures. Blood NEFA were determined by the method of Itaya and Ui [33]. Serum leptin was measured through ELISA kit (R&D Systems, Inc., Minneapolis, MN, USA). Serum glucose concentrations were measured with a glucometer (One Touch UltraSmart; Lifescan, Milpitas, CA, USA) and insulin levels were measured using a rat insulin radioimmunoassay kit (Millipore Corporation, Billerica, MA). HOMA (homeostasis model assessment), an index of IR, was calculated using the formula [HOMA = fasting glucose (mmol/L) × fasting insulin (µU/ml)/22.5]. The hepatic triglycerides were measured by a Triglyceride G-Test kit (Wako Pure Chemical Industries, Osaka, Japan).

### Hepatic Iron Content (HIC)

Hepatic iron content (HIC) was determined as described elsewhere [34]. Liver (1 g) was treated with 65% of nitric acid at 100°C for 30 minutes and then with 60% perchloric acid at 150–180°C for 2 h. After centrifugation (3000 g for 12 minutes) the supernatant was diluted with deionized water and non-heme iron was determined by graphite furnace atomic absorption spectrophotometer (Perkin Elmers 4100 ZL, Italy) and expressed as µg non-heme iron/g of tissue weight.

### Lipid Peroxidation

To determine lipid peroxidation the malondialdehyde (MDA) levels were measured. Briefly, livers were homogenized in 1.15% KCl solution and processed, as previously described [35]. The sample absorbance was measured by spectrophotometry, and MDA values were calculated by comparison with the OD_550_ of standard solutions of 1,1,3,3-tetramethoxypropan 99% malonyldialdehyde bis (dymethyl acetal) 99% (Sigma-Aldrich).

### Preparation of Liver Lysates

Liver lysates for electrophoretic mobility-shift assay and Western blot analysis were prepared from all animals as previously described [19]. Briefly, tissue samples (0.3 g) were disrupted by homogenization on ice in lysis buffer (20 mM Tris–HCl, pH 7.5, 10 mM NaF, 150 mM NaCl, 1% Nonidet P-40 (NP-40), 1 mM phenylmethylsulphonyl fluoride, 1 mM Na_3_VO_4_, leupeptin and trypsin inhibitor 10 mg/ml). After 30 min the supernatant fraction was obtained by centrifugation at 21 000×g for 15 min at 4°C and then stored at −80°C. Protein concentration was determined by the Bio-Rad protein assay (Bio-Rad, Milan, Italy).

### Western Blot Analysis

Protein lysates (50–100 µg) were separated on SDS-PAGE, as previously reported [19]. Blots were probed with anti-nitrotyrosine (dilution, 1∶5000; Millipore, Billerica MA, USA) or anti-FPN-1 (dilution, 1∶1000, kindly provided by K. Pantopoulos from McGill University, Montreal, QC, Canada) [36], or anti-human ferritin (dilution, 1∶1000; Dako Cytomation, Glostrup, Denmark), or anti-human transferrin receptor-1 (dilution, 1∶1000; Zymed Laboratories Inc., CA, USA), or anti-human IRP1 (dilution, 1∶250; Santa Cruz Biotechnology, Inc., Santa Cruz, CA, USA). Subsequently, the membranes were incubated with the appropriate secondary antibodies (Jackson ImmunoResearch Laboratories, Baltimore Pike, West Grove, PA). GAPDH antibody (Sigma-Aldrich, Milan, Italy) was used to normalize the results.

### Electrophoretic Mobility-shift Assay

Plasmid pSPT-fer containing the IRE sequence of the human H-ferritin was transcribed *in vitro* as previously reported [37]. Protein extracts (5 µg) were incubated with *in vitro*-transcribed ^32^P-labelled IRE RNA and the reaction was performed according to a procedure described elsewhere [18]. To recover total IRP1 RNA-binding activity, extracts were pre-incubated with 2-mercaptoethanol (2-ME) at 2% (v/v) final concentration, before the addition of ^32^P-labelled IRE RNA to reveal “latent” IRP1 binding activity. After 6% non-denaturing PAGE, RNA–IRPs complexes were visualized by autoradiography at −80°C and quantified by GS-800 imaging densitometer (Bio-Rad). The results are expressed as the percentage of IRP1 binding activity versus control samples (100% of IRP1 RNA-binding activity) and are the average ± S.E.M. values.

### Real-time PCR

Total RNA, isolated from liver tissue, was extracted using TRIzol Reagent (Invitrogen Biotechnologies), according to the manufacturer’s instructions. cDNA was synthesized using a reverse transcription kit (Maxima First Strand cDNA Synthesized Kit, Fermentas, Ontario, Canada) from 1 µg total RNA. PCRs were performed with an ABIPrism HT7900 fast Real-time PCR System instrument and software (Applied Biosystem). Primer sequences for the targeted rat genes are the following: forward CCAAT/enhancer-binding protein α (C/EBPα), AGCAACGAGTACCGGGTACG; reverse C/EBPα, TGTTTGGCTTTATCTCGGCTC; forward interleukin (IL)-6, ACAAGTGGGAGGCTTAATTACACAT; reverse IL-6, TTGCCATTGCACAACTCTTTTC; forward leptin, TTGTCACCAGGATCAATGACATTT; reverse leptin, GACAAACTCAGAATGGGGTGAAG; forward tumor necrosis factor (TNF)-α, CATCTTCTCAAAACTCGAGTGACAA; reverse TNF-α, TGGGAGTAGATAAGGTACAGCCC; forward ribosomal protein L19 (Rpl19), GAAGGTCAAAGGGAATGTGTTCA; reverse Rpl19, CCTTGTCTGCCTTCAGCTTGT; rat hepcidin primers were synthesized by SABiosciences QIAGEN S.p.A Company (Milano, Italy) catalog #PPR43953A.

The PCR conditions were 10 min at 95°C followed by 40 cycles of two-step PCR denaturation at 95°C for 15 s and annealing/extension at 60°C for 60 s. Each sample contained 1–100 ng cDNA in 2X Power SYBRGreen PCR Master Mix (Applied Biosystem) and 200 nmol/l of each primer (Eurofins MWG Operon, Ebersberg, Germany) in a final volume of 25 µl. The relative amount of each mRNA was normalized to Rpl19 rRNA levels as housekeeping gene, and the data were analyzed according to the 2^−ΔΔCT^ method.

### Statistical Analysis

All data were presented as mean ± SEM. The statistical analysis was performed using Graph-Pad Prism (Graph-Pad software Inc., San Diego, CA) and ANOVA test for multiple comparisons was performed followed by Bonferroni’s test. Statistical significance was set at *P*<0.05.

## Results

### Body and Biochemical Parameters

Weight gain of HFD fed animals did not significantly change after 5 or 8 weeks (HFD 5 weeks169.5±2.38 vs CD 157.4±5.45 g; HFD 8weeks 290.3±7.29 vs CD 267.3±10.22 g), conversely the increase in fat mass raised significance at 8 weeks (HFD 5 weeks 32.18±1.41 vs CD 27.67±2.55 g; HFD 8weeks 62.28±3.5 vs CD 38.52±0.34 g, P<0.001). The evaluation of liver weight revealed no significant variation among groups.

Biochemical serum parameters are reported in table 1. Circulating levels of AST and ALT resulted increased both at 5 and 8 weeks. Moreover, HDL decreased time-dependently, paralleled by the increase of LDL, while triglycerides showed a trend of increase. Interestingly, serum NEFAs were significantly and time-dependently increased by HFD. Serum iron parameters were analyzed and although data were highly variable within the same group, the iron content resulted in a trend of reduction following HFD feeding. This trend was reflected in the decrease of ferritin level observed at 5 weeks, that results attenuated after 8 weeks. Moreover, we observed a time-dependent decrease in TIBC values, related to a not significant trend of reduction in transferrin saturation.

**Table 1:** Changes in serum parameters of rats fed with control (CD) and high fat (HFD) diet for 5 or 8 weeks.

	CD	HFD 5 weeks	HFD 8 weeks
ALT (U/l)	30.4±1.6	47.7±4.6*	52.0±5.2**
AST (U/l)	134.4±6.7	218.8±24.1*	260.3±45.0*
HDL (mg/dl)	67.5±1.1	28.8±0.8***	20.0±1.3*** ^,^^###^
LDL (mg/dl)	30.9±1.8	45.5±2.9**	46.9±2.7**
TGL (mg/dl)	38.2±3.1	46.5±4.0	49.8±2.0
NEFA (µmol/l)	251.7±50.1	508.3±78.1*	765.6±44.3*** ^,^ #
Iron (µg/dl)	127.9±28.0	106.8±13.7	64.4±6.9
Ferritin (ng/ml)	184.7±17.5	137.0±6.6	152.9±16.1
Transferrin iron capacity (TIBC)	241.3±12.4	236.3±7.2	183.9±10.7** ^,^ ##
Transferrin saturation (% )	55.2±12.8	46.6±6.8	36.8±5.7

Values are means ± S.E.M of at least six animals.

* P<0.05,

** P<0.01;

*** P<0.001 vs CD.

# P<0.05.

## P<0.01, and ^###^P<0.001 vs HFD 5 weeks.

### HFD Induces Liver Damage and Insulin Resistance

**Figure 1:**
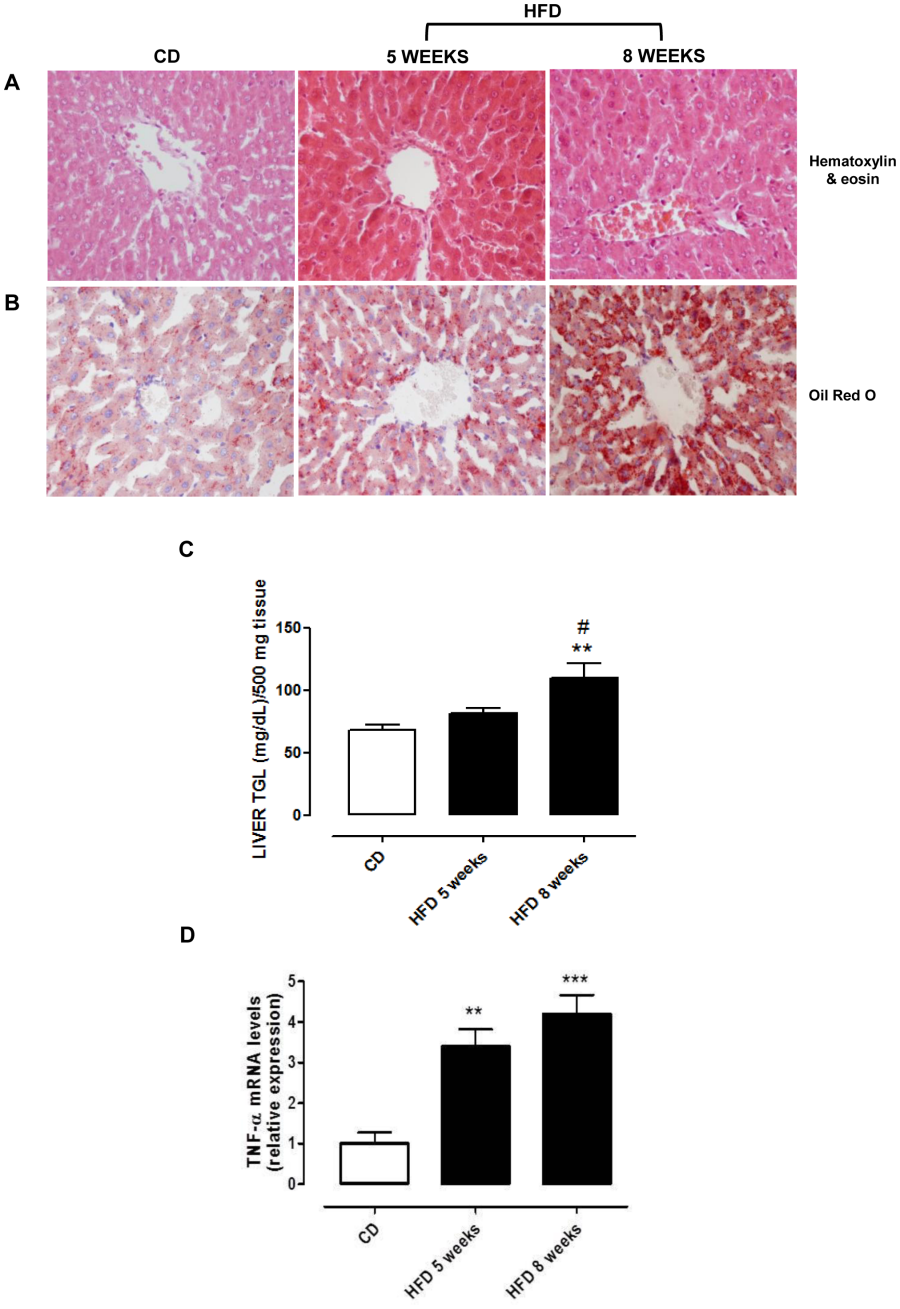
Histological analysis, lipid and TNF-α content in liver of young rats on control (CD) and high fat (HFD) diet for 5 or 8 weeks. Liver sections were stained with (**A**) hematoxylin-eosin or (**B**) Oil red O. Micrographs in both panels are representative pictures with magnification 400 X. (**C**) Quantitative analysis of liver TGL content obtained from all animals is expressed as mg/dl in 500 mg tissue. (**D**) Real time-PCR of TNF-α mRNA is shown. **P<0.01 and ***P<0.001 vs CD; #P<0.05 vs HFD 5 weeks.

Hepatic tissue analysis from rats fed with HFD revealed a progressive increase in steatosis and inflammatory damage in comparison with CD fed animals. Hematoxylin-eosin -stained liver samples obtained from the two groups fed HFD showed initial signs of liver inflammation characterized by the presence of mixed inflammatory cell infiltration and hepatocyte necrosis or apoptosis which appeared throughout the lobule and more evident at 8 weeks (Fig. 1A). As shown in Fig. 1B, HFD rats showed progressive time-dependent steatosis (from grade 2 to grade 3) with a histological pattern characterized by microvesicular steatosis, already evident after 5 weeks of HFD. The hepatocytes showed the cytoplasm filled with small vacuoles which were uniform in size and smaller than the centrally located nucleous. No alterations were observed in the liver of the rats fed with the control diet. To further characterize the progressive steatosis and inflammation, we assayed hepatic triglycerides, which were significantly increased in 8 weeks HFD-fed animals (Fig. 1C), and time dependent increase of mRNA TNF-α content (Fig. 1D). Accordingly, steatosis progression was also related to the increase in HOMA-IR. In fact, after 8 weeks of HFD feeding, HOMA (2.28±0.51) was higher than that at 5 weeks (1.45±0.33) and significant versus control value (0.84±0.14; P<0.05).

### HFD Increases Iron Content and Oxidative Stress in Liver

**Figure 2:**
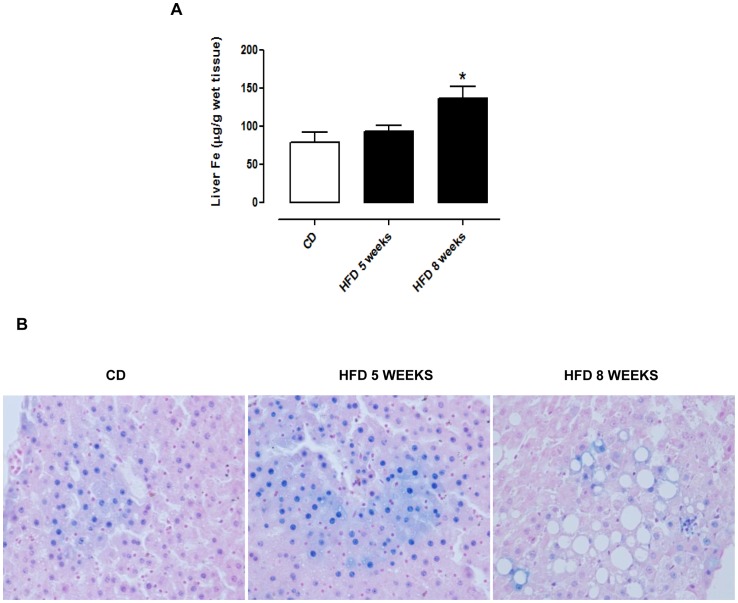
HFD causes iron overload in liver. Tissue iron content was measured by atomic absorption (A) and Perls’blue staining (B) in liver of control diet (CD) or HFD fed rats (5 or 8 weeks). Micrographs in panels are representative pictures with magnification 400 X. The displayed data are mean values ± SEM of rats on CD or HFD. *P<0.05 vs CD.

**Figure 3:**
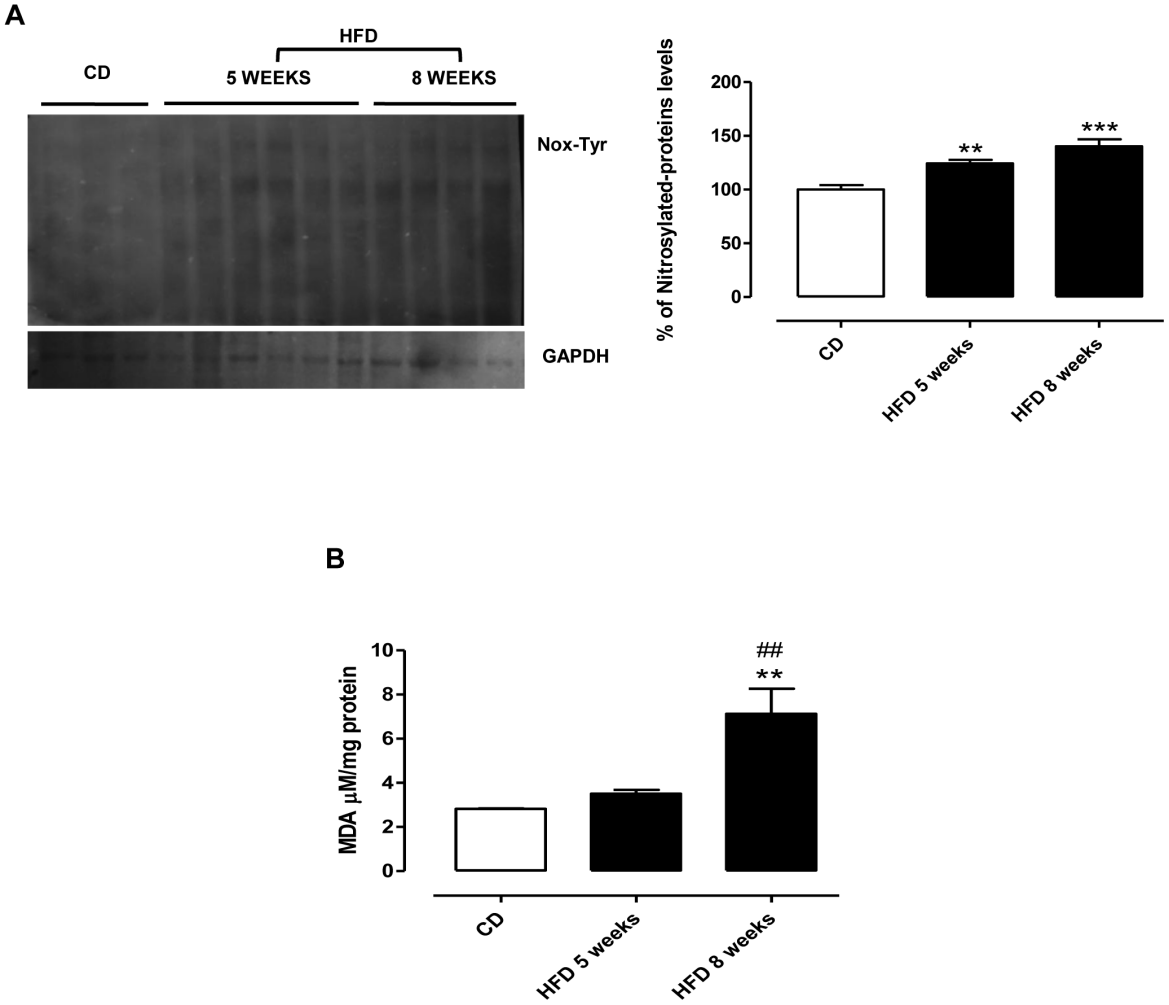
HFD causes oxidative stress in liver. Tissue oxidative stress was evaluated after 5 or 8 weeks feeding with HFD by protein nitrotyrosylation (A) and MDA measurament (B). The displayed data are mean values ± SEM of rats on CD or HFD. **P<0.01, and ***P<0.001 vs CD; ##P<0.01 vs HFD 5 weeks.

To investigate if hepatic inflammation and damage could be related to an increase of hepatic iron content (HIC), we analyzed the non-heme iron by atomic absorption spectroscopy and Perls’ Prussian blue staining. We observed a trend of increase in HIC in HFD group at 5 weeks, which became significant at 8 weeks (Fig. 2A). After 5 and 8 weeks of HFD, an increase of iron content was also observed by Perls’ staining (Fig. 2B) associated to many vacuoles in hepatocytes at 8 weeks. The prolonged feeding with HFD caused an evident oxidative stress, as shown by the time-dependent increase in protein nitrosylation, already significant at 5 weeks (Fig. 3A) and by MDA amount, generated by phospholipids peroxidation, which was markedly increased and significant at 8 weeks (Fig. 3B).

### HFD causes Inflammation, Increases Hepcidin, and Reduces FPN-1 Expression in Liver

**Figure 4:**
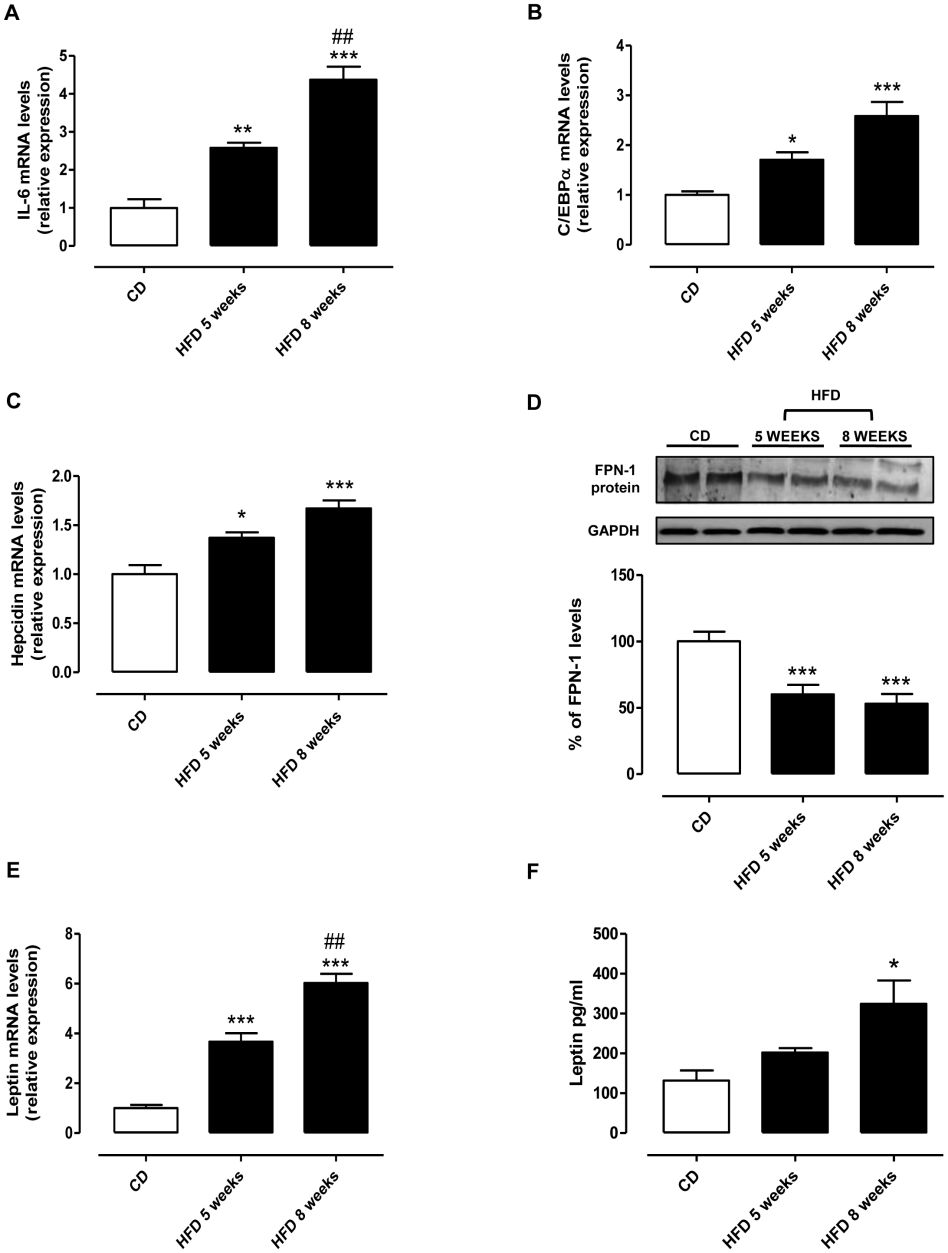
HFD induces pro-inflammatory markers and affects time-dependently c/EBPα, hepcidin, and FPN-1 expression. (A) Real time-PCR of IL-6, (B) c/EBPα and (C) hepcidin mRNA in liver extracts from CD and HFD fed rats for 5 or 8 weeks. (D) Immunoblot of ferroportin (FPN-1) is shown. The bands were densitometrically quantified and plotted. (E) Real time-PCR of hepatic leptin mRNA and (F) serum leptin level, expressed as pg/ml, are shown. *P<0.05; **P<0.01, and ***P<0.001 vs CD; ##P<0.01 vs HFD 5 weeks.

Behind TNF-α content and its increase in steatotic liver, we also evaluated other inflammatory parameters, as IL-6 and leptin. As reported in Fig. 4A and B, IL-6 and C/EBPα mRNA expression significantly increased in a time-dependent manner. As depicted in Fig. 4C, HFD increased hepcidin mRNA at 5 weeks, and more significantly at 8 weeks. Since hepcidin causes FPN-1 internalization and degradation, we measured FPN-1 expression, which was significantly reduced both at 5 and 8 weeks (Fig. 4D). Interestingly, leptin mRNA at hepatic level was also increased, reaching a significant value at 8 weeks (Fig. 4E), that was related to an increase in serum leptin level (Fig.4F).

### HFD Increases IRP1 Activity Affecting Ferritin and TfR-1 Expression in Liver

**Figure 5:**
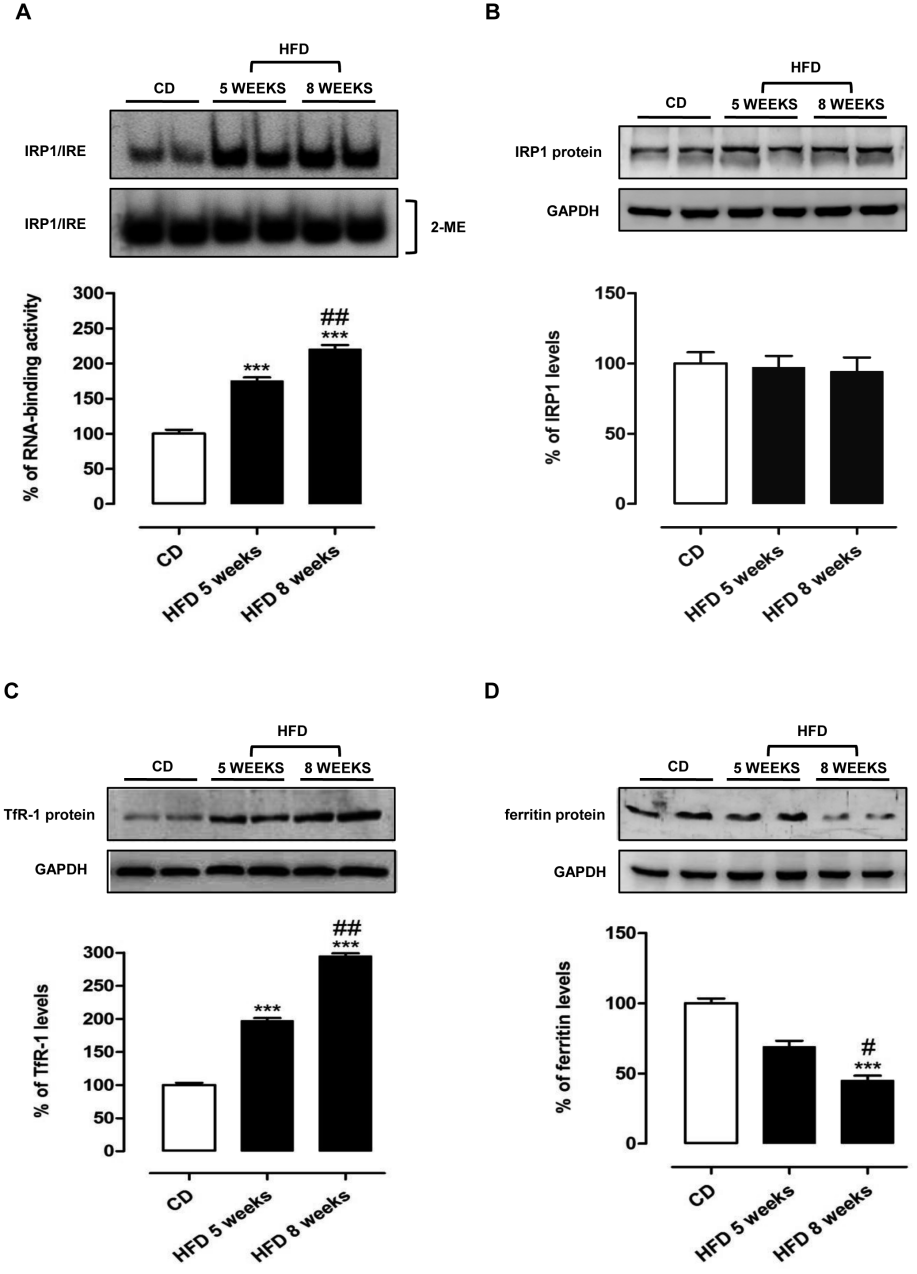
Time dependent modulation of iron metabolism related proteins by HFD. (A) IRP1 RNA-binding activity in liver after control (CD) and high fat (HFD) diet for 5 or 8 weeks. Immunoblots showed IRP1 (B), TfR1 (C), and ferritin (D) protein levels in extracts from liver of rats after CD and HFD. The results of experiments were plotted in a bar graph as percent of the control. GAPDH was used as internal control. Shown are the means ± S.E.M. of all bands plotted in a bar graph compared to control animals. ***P<0.001 vs CD; #P<0.05;##P<0.01 vs HFD 5 weeks.

In order to investigate the effects of HFD-induced steatosis on iron metabolism, we analyzed the RNA-binding activity of IRPs by RNA band-shift assay. As shown in Fig. 5A, HFD caused a remarkable increase in the RNA-binding activity of IRP1. This effect was significant and time-dependent, showing about 2-fold increase *vs* CD already at 5 weeks. To determine the total amount of IRP1 activity, all samples were incubated with mercaptoethanol (2-ME). Concerning IRP1 protein expression, Western blot analysis showed no significant modifications (Fig. 5B), suggesting that the observed modulation of IRP1 activity was not related to protein content. In all samples no IRE-binding activity attributable to IRP2 was detected. In our experimental conditions IRP2-IRE complex could be below detection level, however it is known that IRP1 is the predominant iron regulatory protein in rat liver [38].

Moreover, HFD remarkably increased TfR-1 and reduced ferritin expression when compared with animals on CD. In fact, the TfR-1 content increased of about 2- and 3-fold after 5 and 8 weeks, respectively (Fig. 5C), whereas ferritin expression decreased of about 30 and 60% at 5 and 8 weeks, respectively (Fig. 5D). These results are consistent with the observed RNA-binding activity of IRP1 that leads to an increase of TfR-1 and a reduction of ferritin mRNA translation.

## Discussion

In this study we evidenced that an impairment of iron metabolism and an increase of hepatic iron content, usually associated with NAFLD [39], may play a role in the onset of liver steatosis and progression of the disease.

To evaluate the potential interplay between hepatic iron content, IR, and liver inflammation in the early steps of NAFLD due to fat overnutrition, we administered an high fat diet for short periods (5 and 8 weeks) in young rats, just after weaning, to exclude age and gender influences. Our data evidenced that 5 week feeding with HFD already induced steatosis, as revealed by microvesicular lipid droplets and inflammatory cell infiltration, in association with alteration of lipid profile, increase of transaminases and hepaticTNF-α content. All these modifications were amplified at 8 weeks. The index of IR appeared time-dependently increased, demonstrating a progressive impairment of glucose homeostasis.The influence of iron on the development and progression of NAFLD has been the subject of considerable controversy. It has been reported that exogenous iron overload in steatotic animals aggravated liver injury, switching the experimental steatohepatitis towards the fibrotic stages of the disease [26], [34]. Conversely, other studies do not confirm such an association between hepatic iron content and/or histological iron score and fibrosis [2], [40].

Here, we evaluated, in the early steps of NAFLD, the iron-status parameters and activity and expression of the proteins which have critical functions in iron metabolism. Among these, TfR1 (uptake), ferritin (storage) and FPN-1 (export) are post-transcriptionally regulated by the binding of IRPs to IREs on the respective mRNAs.

In liver of HFD fed animals we found a time-dependent increased activity of IRP1, associated with the increase in TfR1 expression and ferritin down-regulation. These findings were suggestive of an increased iron uptake into hepatocytes, which could lead to an increase of harmful free-iron since ferritin content was reduced. The limited iron-storage capacity makes the cells susceptible to iron-catalyzed ROS damage, contributing to the progression of liver damage. Interestingly, in our experimental conditions the hepatic iron content (HIC) and parameters of oxidative stress resulted significantly increased at 8 weeks.

As a rule, the increase of intracellular iron decreases IRP1 binding activity, however other factors are able to regulate this activity, including oxidative stress [14]. Our apparently contradictory results concerning the increase of IRP1 binding activity, despite the increase of hepatic iron content, can be explained in the light of the following considerations. Previous studies showed that IRP1 binding activity was increased in differentiated adipocytes [37] and this effect was explained by insulin-induced activation of H_2_O_2_-generating system [41]. Similar to insulin, TNF-α acts as a stimulator of NADPH-dependent H_2_O_2_ generation [42]. Our data concerning the increase of TNF-α, protein nitrotyrosilation and lipid peroxidation, observed at 5 weeks and particularly significant at 8 weeks, supports the pivotal role of oxidative stress in IRP1 activation. Moreover, the reduction of ferritin biosynthesis due to the activation of IRP1 by oxidative stress is well documented [14], [43] and our results are consistent with these findings.

In our experimental conditions we show also a reduction of serum ferritin which appears weaken at 8 weeks. The exact function of circulating ferritin is still not entirely clear [44] and under steady state conditions, the serum ferritin level correlates with total body iron stores.

Ferritin plays a crucial role as a cytoprotective and scavenger agent by limiting the reactivity of intracellular iron [18], [37], [45], reducing oxidative stress in acute and chronic inflammatory conditions [46]. In humans, it acts as a buffer against iron deficiency and iron overload, and it is also used as a marker for iron overload disorders, such as hemochromatosis and hemosiderosis in which the ferritin level may be abnormally raised [47], [48]. However, under conditions of oxidative stress, the early degradation of liver ferritin contributes to expand the intracellular free iron pool that, later on, activates multiple molecular mechanisms to reconstitute ferritin content [49].

Serum ferritin levels are commonly elevated in patients with NAFLD because of systemic inflammation, increased iron stores, or both. Kowdley et al [50] examined in human the relationship between elevated serum ferritin and NAFLD severity. They showed that serum ferritin can be considered a marker in identifying patients with NAFLD who are at increased risk of more advanced disease, even among patients without hepatic iron deposition. Furthermore, the same authors indicate that other studies have found conflicting results: some of them reported that serum ferritin levels are associated with increased histological severity and the presence of NASH, whereas others did not. It is unclear whether hyperferritinemia in NAFLD is simply a consequence of disease severity or actively contributes to disease progression in NASH. Interestingly, a recent study has demonstrated that ferritin can act as a proinflammatory cytokine in activated hepatic stellate cells, in an iron independent fashion, inducing different signaling pathways [51].

In our experimental conditions we evidenced a trend of reduction of ferritinemia. We speculate that this difference may be related to the different stage of the disease, in fact our model reflect the onset of the pathology (5–8 weeks HFD, steatosis and mild inflammation without a clear fibrosis) and to the employment of young Sprague-Dawley rats fed with HFD just after weaning. It is well known that young rat have a tendency of rapid growing phase of body and, may be, these animals became easily iron deficient, especially when the diet is mainly based on fatty acids on its calorie source. However, we cannot exclude an rise of serum ferritin in the ongoing and increased gravity of the NASH in our model.

Regarding the increase of hepatic iron content, determined by atomic absorption and Perls’ staining, our data were consistent with the reduction of FPN-1, the main protein involved in iron export.

In addition to post-transcriptional regulation by IRPs, the expression of FPN-1 is also regulated post-translationally through hepcidin-mediated mechanism of degradation. Consistent with FPN-1 down-regulation, we found that hepcidin expression in liver was increased in HFD-fed animals, suggesting an autocrine contribution in the regulation of hepatic iron content.

It is well known that hepcidin acts as a negative regulator in iron homeostasis [52], inhibiting the release of iron recycled from senescent red blood cells by macrophages [53] and the absorption of dietary iron by enterocytes [54].

Hepcidin production is regulated not only by body iron stores, but also by other stimuli [52]. In particular, hepcidin is dramatically augmented in the liver by inflammation and because of chronic disease or infection [21], [22], [55]. *In vitro* studies indicate that IL-6, more than other cytokines (i.e. IL-1α or TNF-α), may be the mediator of hepcidin induction in inflammatory conditions [23]. Other studies demonstrate that in mouse and human, IL-6 is the main cytokine responsible of hepcidin induction and hypoferremia during inflammation and that the IL-6-hepcidin axis is responsible for the hypoferremia in humans [56]. Moreover, in iron deficiency anemia associated with chronic inflammation, the adequate supply of iron to the blood is inhibited by the reduced FPN-1function [22].

On the other hand, IL-6, as well as TNF-α, is a prototypic pro-inflammatory cytokine involved in the earliest events of liver injury, inducing the production of other cytokines that, together, recruit inflammatory cells and trigger a fibrotic process in the liver. In accordance with these findings, we found that HFD increases mRNA expression of these cytokines (IL-6 and TNF-α) in a time-dependent manner, more marked at 8 weeks and related to increased hepatic damage. Moreover, the transcription factor C/EBPα was increased time-dependently by HFD. As well known, this factor regulates hepcidin expression [57] and lipid metabolism [58].

Recently, Ahmed et al. [59] have hypothesized that fat and iron interact to affect body iron status, showing that the iron regulatory genes are activated by macronutrient manipulation in the early stages of NAFLD. However, they lack to demonstrate a significant modulation of hepcidin. In our experimental model (a diet having 58,1% energy derived from fat vs 35% one) we show an HFD-induced increase of hepcidin, in agreement with an increased expression of C/EBPα and IL-6, which are both able to regulate its expression. Therefore, it is conceivable that in our model the raise in hepcidin transcription is at least in part under C/EBPα control, as well as IL-6.

Other recent studies report alterations of iron metabolism in HFD fed animals. In a mouse model of diet-induced obesity and steatosis without inflammation the authors evidenced a decreased serum and tissue iron and an increased serum ferritin. In their model the impaired iron absorption was independent by hepcidin [60]. Moreover, other authors reported that HFD–induced obesity leads to reduced iron storage associated with inflammation [61]. However, no correlation between hepatic iron status and hepcidin mRNA expression was observed in HFD group, indicating that the regulation of hepcidin expression in response to body iron status is perturbed in a prolonged feeding with HFD (16 weeks) associated to significant weight gain and increased adiposity. In our experimental conditions, we found hepatic steatosis and inflammation, without significant modifications in body weight and fat content. We used a different high fat diet which causes an inflammatory steatosis characterized by elevated cytokines and adipokines. Moreover, we show an increase in hepatic iron and related oxidative stress together with an increase in hepcidin transcription and related decrease of ferroportin. However, many other studies are consistent with our results evidencing a significant increase in hepcidin associated to inflammation and often to hypoferremia [62]–[63].

Also leptin, an adipose-derived cytokine, is thought to participate in the onset and progression of IR and NAFLD. Here, we demonstrated a time-dependent increase of leptin mRNA expression in liver and, interestingly, a raise of serum leptin, reaching a significant value at 8 weeks. In liver injury, leptin has a proinflammatory role and is considered to be an essential mediator of liver fibrosis [64].

Liver inflammatory damage, induced by HFD, leads to increased production of cytokines and leptin, which in turn contribute to increased hepatic hepcidin production. This latter event, coupled to the concomitant increased RNA-binding activity of IRP1, causes a decrease in FPN-1, resulting in inhibition of iron release into the blood. On the other hand, the increased IRP1 activity induces TfR-1up-regulation and reduces the expression of ferritin in liver, contributing to the increase in free iron and oxidative stress.

Taken all together, our data clearly show an early impairment of iron metabolism in the initial stage of steatosis contributing to the progression of the disease and the concept of “multiple hits” in the progression of inflammation in NAFLD [1], might include the early alteration of iron metabolism.
